# The effectiveness of nurse-led palliative care needs assessment on patients’ quality of life and symptom burden: a systematic review

**DOI:** 10.1016/j.ijnsa.2025.100343

**Published:** 2025-05-01

**Authors:** Gursharan K. Singh, Claudia Virdun, Megan Rattray, Roslyn Prichard, Rochelle Wynne

**Affiliations:** a Centre for Healthcare Transformation, Faculty of Health, Queensland University of Technology (QUT), Brisbane, QLD, Australia; b Cancer and Palliative Care Outcomes Centre, School of Nursing, Queensland University of Technology (QUT), Brisbane, QLD, Australia; c Flinders Research Centre for Palliative Care, Death, and Dying, College of Nursing and Health Sciences, Flinders University, Bedford Park, SA, Australia; d College of Medicine and Public Health, Flinders University, Adelaide, SA, Australia; e School of Health, University of Sunshine Coast, Sippy Downs, QLD, Australia; f School of Nursing & Midwifery, Centre for Quality & Patient Safety in the Institute for Health Transformation, Deakin University, Geelong, VIC, Australia; g Western Health, St Albans, VIC, Australia

**Keywords:** Nursing, Palliative care, Needs assessment, Quality of life, Symptom burden, Systematic review

## Abstract

**Background:**

Optimal palliative care requires a correct assessment of needs. Nurses are well-placed to undertake this task, but the effectiveness of nurse-led palliative care needs assessment remains uncertain.

**Aim:**

To evaluate the evidence regarding the impact of nurse-led palliative care needs assessment on adult patients with oncological and non-oncological illnesses quality of life, symptom burden and hospitalisations.

**Design:**

Systematic review. The review was registered on the international Prospective Register of Systematic Reviews (PROSPERO) (registration number CRD42023429259).

**Data sources:**

Databases searched were CINAHL, PubMed, Embase, and MEDLINE from inception to April 2024.

**Methods:**

A systematic review of English language, randomised controlled trials, conducted in May 2023, and updated in April 2024, on the impact of nurse-led palliative care needs assessment was undertaken. Two independent reviewers screened papers, and two reviewers independently conducted data extraction and risk of bias assessment using the Cochrane Risk of Bias 2 tool. The data were analysed using a narrative synthesis approach by combining studies according to the outcomes of interest.

**Results:**

Six trials were included, involving oncological patients (*n* = 4), non-oncological patients (*n* = 1) and deceased aged-care residents (*n* = 1). Two studies had ‘low’ risk of bias, two had 'some concerns,' and two had 'high concerns.' There was heterogeneity in the needs assessment tools used and the outcome measures assessed. Researchers who conducted a nurse-led and social worker-led trial in non-oncological patients demonstrated statistically significant improvements in patient quality-of-life and symptom burden. Researchers in two trials found no difference, and two others reported statistically non-significant improvements in quality of life and symptom burden. One group of researchers found no difference in hospitalisations at 6 months. No studies evaluated the inpatient length of stay.

**Conclusion:**

There is a paucity of high-quality evidence on the effectiveness of nurse-led palliative care needs assessments. Future researchers must identify what level of needs predicts poor quality of life, assess interventions tailored to local contexts, and determine how best to evaluate their impact using clinically relevant outcome measures.

**What is already known about the topic**

• Accurate assessment of palliative care needs is essential for effective implementation of palliative care for populations with life-limiting illnesses.
• Nurses are well placed to assess and triage patients and carers with unmet needs, so evidence to support the effectiveness of nurse-led assessment is crucial.

Alt-text: Unlabelled box

**What this paper adds**

• There was conflicting evidence substantiating the effectiveness of nurse-led approaches to palliative care needs assessment.
• Evidence available at the time of this review was hampered by poor quality study designs and variable end-point measures that are not well defined.
• Additional research is urgently needed on the impact of nurse-led palliative care needs assessment on outcomes for patients with non-oncological illnesses.

Alt-text: Unlabelled box

## 1 Introduction

Addressing palliative care needs of patients and their families living with life-limiting illnesses is a significant global challenge (Hui et al., 2022). Palliative care, as defined by the World Health Organisation, is an ‘approach to care which aims to improve the quality of life of patients and families facing issues associated with a life-limiting illness, through prevention and relief of suffering by means of early identification, correct assessment and treatment of pain and of other physical, psychological and spiritual concerns’(World Health Organization, 2020). The accurate assessment of unmet need across populations living with life-limiting illnesses is essential for effective implementation of palliative care (ElMokhallalati et al., 2020). A palliative care needs assessment should include a holistic assessment of physical, psychological, spiritual, and social concerns (Cherny et al., 2015). Other concerns that are included in a palliative care needs assessment are existential needs (Richfield and Johnson, 2020), activities of daily living, information, and sexual and healthcare needs (Rimmer et al., 2022)

Various tools have been developed and tested to assess palliative care needs across different diseases and settings (Cherny et al., 2015). These assessments aid in identifying and facilitating actions to address gaps in care and support, discussing and recording care preferences, and implementing strategies and services to improve outcomes (Remawi et al., 2021). Most of the work on developing and validating needs assessment tools has focused on the needs of patients with cancer, with fewer tools developed for non-oncological conditions, such as dementia, chronic obstructive pulmonary disease, and heart failure (Cherny et al., 2015). The routine implementation of an integrated needs-based model of palliative care, informed by palliative care needs assessment and tailored care planning, remains elusive (Cherny et al., 2015). Given population ageing and increasing rates of palliative care needs in people living with life-limiting illnesses (Moens et al., 2014), the need to embed routine screening of unmet needs is urgent.

Assessing patients is an essential nursing responsibility, making nurses ideally placed to assess and triage patients and carers with unmet needs to the most appropriate health professional or service (Higginson and Jones, 2009). Nurse-led services, defined as a registered nurse leading a service with primary responsibility for a cohort of patients, demonstrated equivalent or better outcomes in terms of health-related quality of life compared to physician-led care or standard care for managing chronic conditions (Chan et al., 2018). Taking into account the demographic imperatives of an ageing population and the limited availability of health professionals with specialist palliative care training to meet these needs (Capurro et al., 2014), integrating nurse-led palliative care needs assessments, triage, and care planning, where nurses have a primary responsibility for assessing all or specific needs, must be considered to ensure individuals with both oncological and non-oncological illnesses receive the right care at the right time. This includes nurses retaining a primary responsibility and leading a specific aspect of a needs assessment, even if other health professionals lead other aspects of the needs assessment. As such, determining the effectiveness of nurse-led palliative care needs assessment is crucial and will help guide the implementation of an optimal model of interdisciplinary palliative care.

Previous researchers have conducted systematic reviews that examined the effect of nurse-led palliative care on outcomes in patients with cancer (Li et al., 2024) and in patients with chronic obstructive pulmonary disease (Ora et al., 2019). Other researchers have conducted a systematic review that examined the costs and cost-effectiveness (Salamanca-Balen et al., 2017); however, no systematic review has examined the evidence of nurse-led palliative care assessments in oncological and non-oncological illnesses. The aim of this systematic review was to evaluate the existing evidence regarding the impact of nurse-led palliative care needs assessment on adult patients with oncological and non-oncological illnesses’ quality of life, symptom burden, and hospitalisation. The research questions this review aimed to answer were:

1. What is the impact of nurse-led palliative care needs assessment on patient quality of life?
2. What is the impact of nurse-led palliative care needs assessment on patient symptom burden?
3. What is the impact of nurse-led palliative care needs assessment on unplanned readmission rate and length of in-patient stay?

## 2 Methods

### 2.1 Design

A systematic review was conducted, and the review protocol was registered on the international Prospective Register of Systematic Reviews (PROSPERO) registration number CRD42023429259.

### 2.2 Eligibility criteria

The study selection criteria were pre-determined by the Population, Intervention, Comparison and Outcome (PICO) framework (Richardson et al., 1995). Randomised controlled trials, reporting the primary analysis (i.e., the original findings of the randomised control trial), published in English, or mixed methods papers where the quantitative component was a randomised control trial were eligible for inclusion. Studies with adult patients (≥18 years old) with oncological or non-oncological illnesses (P) that investigated the impact of nurse-led palliative care needs assessment (I) compared to a ‘usual care’ control group (C) on patient quality of life and symptom burden including symptom severity and frequency (O) were included. Secondary outcomes examined were unplanned readmission and length of stay. The intervention of interest was nurse-led palliative care needs assessment with the aim of identifying patients in need of palliative care (Janssen et al., 2018) or evaluating needs for triage and tailored care planning (O'Connor, 2007).

Studies were excluded if they were conducted among individuals aged 18 years or younger. We also excluded randomised control trial papers presenting a subgroup analysis or secondary analysis, non-experimental quantitative studies (e.g., case-control studies), qualitative studies, quasi-experimental studies, studies using registry data, cross-over randomised control trials assessing two or more interventions (due to the risk of contamination), literature reviews, commentaries, editorials, and non-peer reviewed sources including grey literature, theses, and dissertations.

### 2.3 Search strategy

The following databases were searched: Cumulative Index to Nursing and Allied Health Literature (CINAHL), PubMed, Excerpta Medica Database (Embase) (via the OVID platform), and Medical Literature Analysis and Retrieval System Online (MEDLINE). Both Pubmed and MEDLINE were searched to reduce selection bias. While PubMed can be used to search MEDLINE (as well as other content), MEDLINE allows one to perform a more focused search, leading to different results by searching in each database. The search was not restricted by date to ensure a comprehensive and exhaustive search and to ensure all relevant clinical trials were included. The search was conducted in May 2023 (and updated in April 2024) and was limited to records published in English. Manual searches were conducted by examining reference lists of included studies. The search strategy is included in the Supplementary Material. The search strategy was developed in collaboration with a nursing librarian experienced in conducting systematic reviews.

### 2.4 Study selection

Records from each database were imported into reference management software and then uploaded into Covidence®, a platform that blinds the study selection process and brings together researchers to complete screening and data extraction (Babineau, 2014). Duplicates were removed in Covidence®, and three reviewers (RW, GKS and MR) independently screened the titles and abstracts, so that each record was screened by two reviewers, with a third reviewer (CV) available to moderate disagreements to reach consensus. Following title and abstract screening, papers identified for full-text review were screened independently, with one reviewer (MR) screening all papers and three reviewers (GKS, CV and RW) screening an equal number of full text papers as a second reviewer. A third reviewer (either GKS, CV and RW) was available to moderate disagreements to reach consensus for full-text review.

### 2.5 Data extraction and synthesis

Three reviewers (GKS, MR, CV) extracted data from eligible studies. Data were extracted into a table that included authors; country of origin; year of publication; demographic characteristics of study participants; study methodology; intervention information; details of the comparator group; key findings, including the quality of life instrument and score; symptom burden instrument and score; unplanned readmissions; and author identified strengths and limitations. Author identified strengths and weaknesses were extracted to provide context to the papers and the possible reasons, if any, for why an intervention did not detect a statistically significant difference**.** Each extracted study was cross-checked by two reviewers to ensure accuracy. The primary author of one study was contacted, as there was data which couldn’t be deduced from graphs. The author provided the score for each symptom sub-scale pre- and post-intervention. The primary author of another study was contacted, as there was no information relating to the change in scores from baseline to follow-up for the intervention and usual care group, who confirmed these findings were not available. The reported mean (95 % confidence interval [CI]) scores at baseline and post-intervention and the baseline adjusted mean difference (95 % CI) was extracted for study outcomes. The extracted data were synthesised by combining studies according to the outcomes of interest, which were quality of life, symptom burden, and unplanned hospitalisations and length of stay. There were no criteria used to prioritise the reporting of one study’s findings over others, such as risk of bias. However, findings from adequately-powered studies were noted. Due to heterogeneity in the outcomes and outcome measures and bias in the measurement and selection of reported results, a meta-analysis was not feasible, and a narrative synthesis with a focus on common key findings is presented instead.

### 2.6 Risk of bias assessment

Risk of bias assessment was undertaken by three reviewers (GKS, MR, CV), with each paper reviewed by two reviewers to ensure accuracy. The Cochrane Risk of Bias 2 (RoB 2) tool was used to assess quality assessment and risk of bias for each included study (Sterne et al., 2019), as risk of bias tools can be used for quality assessment (Furuya-Kanamori et al., 2021). This tool is structured into domains of bias, focusing on aspects of trial design, conduct, and reporting aspects (Sterne et al., 2019). A set of questions within each domain aids in gathering information relevant to risk of bias. Depending on the answers obtained from using the tool, the trials were categorised as 'high' to 'low' risk of bias (see Results section).

## 3 Results

### 3.1 Study selection

**Fig. 1:**
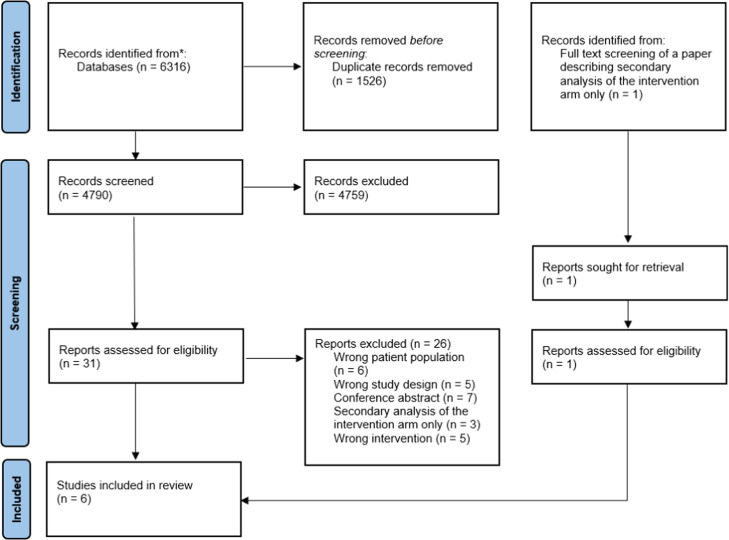
Study identification, screening and inclusion process.

From our search, we identified 6316 records. After duplicates were removed, title and abstract screening was conducted on 4790 papers, resulting in the exclusion of 4759 papers. We then screened the full text of the remaining papers. An additional paper was included that was identified from an excluded paper, which reported on a secondary analysis of the intervention arm only. Ultimately, six papers met the eligibility criteria and were included by consensus agreement (Fig. 1).

### 3.2 Study characteristics

There were four multi-site randomised control trials (Bekelman et al., 2024; Reinke et al., 2022; Schenker et al., 2021; Van den Block et al., 2020) and two single-site randomised control trials (Bjerring et al., 2020; Zimmermann et al., 2023). The trials were conducted in various countries, including the United States of America (*n* = 3) (Bekelman et al., 2024; Reinke et al., 2022; Schenker et al., 2021), Denmark (*n* = 1) (Bjerring et al., 2020), and Canada (*n* = 1) (Zimmermann et al., 2023), while one was conducted across multiple countries, including Belgium, England, Finland, Italy, The Netherlands, Poland, and Switzerland (Van den Block et al., 2020). Detailed study characteristics are presented in Table 1. Participant cohorts were mainly oncological patients (*n* = 4), non-oncological patients (*n* = 1), or deceased residents in aged-care homes (*n* = 1). The number of participants ranged from 79 (Bjerring et al., 2020) to 672 (Schenker et al., 2021) across studies. The studies took place in diverse settings, including the home (Bekelman et al., 2024; Bjerring et al., 2020; Reinke et al., 2022), aged-care homes (Van den Block et al., 2020), an outpatient oncology practice (Schenker et al., 2021), and a palliative care clinic (Zimmermann et al., 2023).

**Table 1:** Study characteristics.

Autor(s) (year), Country	Study design	Setting & sample	Needs assessment tool, intervention description & comparator	Key findings		
				Quality of life instrument and score	Symptom burden instrument and score	Unplanned readmissions
Bekelman et al. (2024) United States	Multi-site RCT	Participants home Total: 306 patients (10 % F) with a diagnosis of COPD, HF, ILD at 2 Veterans Administration health system in the top 20th percentile risk for hospitalisation/death and had a poor quality of life and symptom burden. IG: *n* = 152, 68.87 (8.04)^a^ y CG: *n* = 154, 68.88 (7.42)^a^ y	Selection of initial symptom and structured symptom assessment scale. IG: A registered nurse addressed symptoms and a social worker provided structured counseling. The intervention consisted of six phone calls each by the nurse and social worker, planned 2x a month. Patients could select an initial symptom to target, and the nurse assessed and managed symptoms using structured guidelines, implemented team recommendations and reassessed symptoms using a rating scale, identified an activity goal with the patient and helped with barriers to progress and provided disease education. The social worker conducted an initial psychosocial assessment and provided counselling calls. The nurse and social worker met weekly with the physicians as needed and ordered tests and medication if needed alongside the primary care clinician. CG: Care from pulmonology, cardiology, mental health and palliative care. CG participants were given an educational handout developed for the study and detailed self-care for their respective illnesses. Patients in CG who had significant depressive symptoms were notified and their primary care clinician.	FACT-G^c^ At 6 m, the mean quality of life score improved 6 points in the intervention arm compared to 1.4 points in the usual care (difference 4.6 points [95 % CI: 1.8,7.4) *p=* 0.001). IG: Baseline (Mean [SE]): 52.9 [4.0] 6 m: 58.9 [4.0] Mean [SE] change from baseline: 6.0 [1.0] CG: Baseline (Mean [SE]): 52.7 [4.0] 6 m: 54.1 [4.0] Mean [SE] change from baseline: 1.4 [1.0]	PHQ-8,^d^ GAD-7^c^ At 6 m, mean depression score reduced by 2.42 points in the intervention arm compared to 0.06 points in usual care (difference -2.36 points [95 % CI: -3.52,1.21] *p* < 0.001. At 6 m, mean anxiety score reduced by 2.11 point in the intervention group compared to 0.27 points in usual care (difference -2.39 points [95 % CI, -3.53, -1.25] *p* < 0.001). IG: PHQ-8 Baseline (Mean [SE]): 12.45 [1.41] 6 m: 10.02 [1.43] Mean [SE] change from baseline: -2.42 [0.42] IG: GAD-7 Baseline (Mean [SE]): 7.93 [1.42] 6 m: 5.82 [1.43] Mean [SE] change from baseline: -2.11 [0.41] CG: PHQ-8 Baseline (Mean [SE]): 11.64 [1.40] 6 m: 11.58 [1.42] Mean [SE] change from baseline: −0.06 [0.42]. CG: GAD-7 Baseline (Mean [SE]): 6.65 [1.41] 6 m: 6.92 [1.42] Mean [SE] change from baseline: 0.27 [0.41]	At 6 m there was no difference between intervention and usual care groups among patients who had been hospitalised (*p* = 0.45). IG: Hospitalised at all: 33 CG: Hospitalised at all: 26
Bjerring et al. (2020) Denmark	Single-site RCT	Participants home Total: 79 patients (16 % F) treated with self-expandable metal stents for malignant dysphagia due to incurable oesophageal or gastroesophageal junction cancer IG: *n* = 39, 74 (49–95) y^b^ CG: *n* = 40, 71 (51–86)^b^ y	A checklist including patient status, nutritional screening, pain screening and treatment using visual analogue scale, nausea and vomiting, fatigue, social problems etc. IG: Patients offered "open admission" to department with a Hot-Line phone number. Home visits included advice and assessment of patient's needs; physicians consulted for treatment changes. Patients in intervention arm had prescheduled home visits by dedicated nurse after 1 and 6 weeks, plus a call after 11 weeks. The same nurse conducted all visits. Basic checklist for potential topics, but visits focused on most relevant needs. Nurse completed structured report after each visit or call, no specific protocol otherwise. CG: Usual follow-up (details not reported)	EORTC QLQ-C30, EORTC QLQ -OES18 The pre- self-expandable metal stents global quality of life scores were 35 and 33 (*p* = 0.61) for the intervention group and standard group, respectively. Seven weeks after self-expandable metal stent insertion a trend towards improved global health related quality of life scores was seen in the intervention group (48 vs. 38, respectively), which did not reach statistical significance (*p* = 0.13).	EORTC QLQ-C30 (fatigue, pain, nausea and vomiting) The symptom scales displayed generally lower scores in those belonging to the intervention arm at 12 weeks, but these were not statistically significant. IG: Dysphagia score pre-intervention:42 Dysphagia score post-intervention:68 Fatigue score pre-intervention:55 Fatigue score post-intervention:39 Pain score pre-intervention:38 Pain score post-intervention:24 Nausea score pre-intervention: 35 Nausea score post-intervention: 17 CG: Dysphagia score pre-intervention: 39 Dysphagia score post-intervention:55 Fatigue score pre-intervention: 63 Dysphagia score post-intervention:55 Pain score pre-intervention:39 Pain score post-intervention:28 Nausea score pre-intervention: 39 Nausea score post-intervention: 21	Not reported
Reinke et al. (2022) United States	Multi-site RCT	Participants home Total: 151 patients (2 % F) with a diagnosis of lung cancer (within 8 weeks) or a recurrence of primary lung cancer within 5 years, >40 years of age IG: *n* = 73, 69.0 (8.6)^a^ y CG: *n* = 78, 70.0 (6.4)^a^ y	An evidence-based End-of-Life Nursing Education Consortium protocol for structured assessment of common symptoms including pain, dyspnea, fatigue, cough, anxiety, depression (first 2 questions of PHQ-9), and gastrointestinal complaints and a psychosocial needs assessment using “Needs Assessment Tool.” IG: The intervention was based on the Chronic Care Model, incorporating symptom management, person centred care plans and education on lung cancer symptoms and consisted of 9 phone calls over 3 months (weekly for 4 weeks then every other week for 8 weeks). The nurses met with the study team weekly to address participant/nurse concerns and to assess intervention fidelity. The nurse documented changes in symptoms in the electronic health record and made specific recommendations to address symptom, coordinate care or change medications which was communicated to the clinician. CG: Usual care (details not reported)	FACT-L Change in score for FACT-L from baseline to 3 months was not reported for IG and CG.	FACT-L LCS Change in score for FACT-L LCS from baseline to 3 m was not reported for IG and CG.	Not reported
Schenker et al. (2021)United States	Multi-site RCT	17 Outpatient oncology practices Total: 672 patients (54 % F) with metastatic solid tumors who were undergoing oncological care and oncologist “would not be surprised if the patient died in the next year” IG: *n* = 336 CG: *n* = 336 69.3 (10.2)^a^ y combined groups	ESAS and DT IG: Infusion room nurses were trained to focus on symptom management, emotional support, advance care planning, and coordination. Visits, with the same nurse, preceded or followed regular oncology appointments, including phone consultations. Nurses used checklists for goals and tips. Initial visits prioritised rapport, symptom management, and surrogate decision-making. Subsequent visits covered treatment preferences and advance directives. Visits responded to patient-reported symptoms. Nurses collaborated on shared care plans with patients and caregivers. After each visit, nurses informed the patient's oncologist and conducted follow-up calls to address plan-related issues. CG: Usual care - oncology care according to best practices, including all supportive measures deemed appropriate by oncology team	FACIT-Pal^c^ At 3 m, no difference in mean [SD] quality-of-life score was found between the intervention and standard care groups (130.7 [28.2] vs 134.1 [28.1]; adjusted mean difference, 1.20; 95 % CI, −2.75 to 5.15; p = .55). IG: Mean [SD] Pre: 127 [24.9] 3 m: 130.7 [28.2] CG: Mean [SD] Pre: 133 [25.8] 3 m: 134.1 [28.1]	ESAS, HADS^d^ There was no difference between groups in 3 m mean [SD] symptom burden (23.2 [16.6] vs 24.0 [16.1]; adjusted mean difference, −2.64; 95 % CI, −5.85 to 0.58; *P* = .11) or mood symptoms (HADS depression subscale score: 5.1 [3.4] vs 4.8 [3.7], adjusted mean difference, −0.08 [95 % CI, −0.71 to 0.57], *p* = .82; HADS anxiety subscale score: 5.7 [3.9] vs 5.4 [4.2], adjusted mean difference, −0.31 [95 % CI, −0.96 to 0.33], *p* = .34]). IG: ESAS total score (Mean [SD]) Pre: 26.3 [16.0] Post: 23.2 [16.6] IG: HADS: Depression subscale score (Mean [SD]) Pre: 5.70 [3.93] Post: 5.1 [3.4] IG: HADS: Anxiety subscale score (Mean [SD]) Pre: 6.28 [4.07] Post: 5.7 [3.9] CG: ESAS total score (Mean [SD]) Pre: 24.2 [15.9] Post: 24.0 [16.1] CG: HADS: Depression subscale score (Mean [SD]) Pre: 5.12 [3.56] Post: 4.8 [3.7] CG: HADS: Anxiety subscale score (Mean [SD]) Pre: 5.28 [3.66] Post: 5.4 [4.2]	Not reported
Van den Block et al. (2020) Belgium, England, Finland, Italy, the Netherlands, Poland, and Switzerland	Multi-site RCT	78 Aged-care homes Total: 551 (64 % F) deceased residents IG: *n* = 279, 85.22 (9.13)^a^ y CG: *n* = 272, 85.68 (9.00)^a^ y	A Mapping Changes in Condition chart IG: Assessment, Care Planning, and Review of resident needs and problems. The 'Mapping Changes in Condition chart' tool was utilised monthly by nurses and care assistants to track changes in a resident's physical condition, aiding staff in recognising changes over the months. By completing this process every month (and weekly during a resident's final phase of life), the trajectory of the resident's condition was observed over time. CG: Usual care (details not reported)	Not reported	EOL-CAD EOLD-CAD total score reported by staff did not differ between intervention and control groups (baseline-adjusted mean difference, −0.55; 95 % CI, −1.71 to 0.61; *p* = .35) IG: Score (Mean, [95 %CI]) Baseline:30.64 [29.33–31.95] Post-intervention:30.86 [20.61 to 32.12] CG: Score (Mean, [95 %CI]) Baseline: 30.22 [28.92 to 31.53] Post-intervention: 31.00 [29.77 to 32.23]	Not reported
Zimmermann et al. (2023) Canada	Single-site RCT	Palliative care clinic (*n* = 1) Total: 69 patients (62 % F) with advanced solid tumors and an oncologist-estimated prognosis of 6–36 m IG: *n* = 33, 64.0 (25.0–79.0)^a^ y CG: *n* = 36, 64.0 (39.0–87.0)^a^ y	Not mentioned but refers to assessment of symptoms, psychological distress, social support and home services IG: In-person consultation within 1–2 weeks. Comprehensive symptom assessment following provincial guidelines. Monthly follow-up in clinic for 6 m (or more if requested). Access to 24-hour on-call service for urgent issues. Assessment for home nursing care and arrangement of services. Transfer to home palliative care team if ECOG performance status ≥ 3 or patient prefers home care. Dedicated inpatient beds for urgent symptoms and end-of-life care. CG: Usual oncology care - routine ESAS-r screening at outpatient visits, with nurses reviewing and discussing scores with patients, then informing oncologists for potential interventions/referrals	FACT-G7^c^There was less deterioration in quality of life at 6 m from baseline in the intervention arm versus usual care (Mean change score −0.2 [95 % CI: −2.9, 2.4] versus −2.1 [95 % CI: −4.4, 0.3], respectively), but the difference was not statistically significant. IG: Baseline (Mean [SD]): 18.5 [5.1] 2 m Observed change from baseline mean [95 % CI]: 1.0 [-3.1, 1.0] 4 m: -1.7 [-4.1, 0.6] 6 m: -0.2 [-2.9, 2.4] CG: Baseline (Mean [SD]): 19.7 [4.3] 2 m Observed change from baseline mean [95 % CI]: -3.1 [-4.7, -1.5]4 m: -2.4 [-4.2, -0.7] 6 m: -2.1 [-4.4, 0.3]	ESAS-r-CS TDS, PHQ-9Symptom burden tended to be better in intervention, but did not reach statistical significance at 6 m (difference in change scores between intervention and usual care: -5.51; 95 % CI -14.29 to 3.27, *p* = 0.39)IG: ESAS-r-CS TDS: Baseline (Mean [SD]): 20.7 [16.9] 2 m Observed change from baseline mean [95 % CI]: 3.4 [-4.4, 11.1]4 m: 5.7 [-2.7, 14.0]6 m: 3.7 [-4.0, 11.3]IG: PHQ-9:Baseline (Mean [SD]): 4.4 [4.3]2 m Observed change from baseline mean [95 % CI]: 0.2 [-1.9, 2.3] 4 m: 1.4 [-0.7, 3.6] 6 m: 0.5 [-2.0, 3.0]CG: ESAS-r-CS TDS:Baseline (Mean [SD]): 20.0 [13.1]2 m (Observed change from baseline mean [95 % CI]: 3.4 [−0.3, 7.1]4 m: 7.5 [2.2, 12.8]6 m: 8.4 [1.5, 15.3]CG: PHQ-9Baseline (Mean [SD]): 4.9 [3.6]2 m (Observed change from baseline mean [95 % CI]: 0.9 [0, 1.8]4 m: 1.9 [0.2, 3.6]6 m: 1.7 [-0.3, 3.7]	Not reported

CG: Control Group; CI: Confidence Interval; COPD: Chronic obstructive pulmonary disease; DT: Distress Thermometer; ECOG: Eastern Cooperative Oncology Group; EOL-CAD: End-of-Life in Dementia Comfort Assessment while dying; EORTC QLQ-C30: European Organization for the Research and Treatment of Cancer Quality of Life -Core 30 Questionnaire; EORTC QLQ -OES18: European Organization for the Research and Treatment of Cancer Quality of Life -Core Questionnaire -Oesophageal 18; ESAS: Edmonton Symptom Assessment Scale; ESAS: Edmonton Symptom Assessment Scale – revised; ESAS-r-CS TDS: Edmonton Symptom Assessment Scale – revised – Constipation and Sleep Total Distress Score; F: Female; FACIT-Pal: Functional Assessment of Chronic Illness Therapy-Palliative care; FACT-G: Functional Assessment of Cancer Therapy–General; FACT-G7 scale: Abbreviated version of the Functional Assessment of Cancer Therapy–General (FACT-G) scale; FACT-L: Functional Assessment of Cancer Therapy - Lung Scale; FACT-L LCS: Functional Assessment of Cancer Therapy - Lung Scale Lung Cancer Subscale; GAD-7: Generalized Anxiety Disorder 7 items; HF: Heart failure; HADS: Hospital Anxiety and Depression Scale; IG: Intervention group; ILD: Interstitial lung disease; m: month(s); p: probability; PHQ-9: Patient Health Questionnaire – 8 items; PHQ-9: Patient Health Questionnaire – 9 items; RCT: Randomised control trial; SD: Standard deviation; SE: standard error; y: years.

a Age reported as mean and (SD) unless otherwise specified:.

b Age reported as median and (range).

c Higher scores indicate better quality of life.

d Higher scores indicating greater symptom burden, anxiety and depression.

The needs assessment tools used by nurses included a Mapping Changes in Condition chart (Van den Block et al., 2020), the Edmonton Symptom Assessment Scale and Distress Thermometer (Schenker et al., 2021), an evidence-based End-of-Life Nursing Education Consortium protocol (Reinke et al., 2022), and a nurse checklist of patient status, nutritional and pain screening, nausea, vomiting, fatigue, and social problems (Bjerring et al., 2020). Zimmermann et al. (2023) did not refer to the specific needs assessment tool used, stating that a comprehensive assessment of symptoms, psychological distress, social support, and home services was undertaken, and other researchers (Bekelman et al., 2024) stated that participants could select an initial symptom. This was assessed with a structured symptom scale, although the specific scale was not described. The timing of outcome data collection varied among studies. Only one study reported collecting data beyond 6 months, at 13 and 17 months (Van den Block et al., 2020). Two studies reported data up to 6 months, at 6 months (Bekelman et al., 2024), and at 2, 4 and 6 months (Zimmermann et al., 2023). Three studies reported data up to 3 months, at baseline and at 3 months (Reinke et al., 2022; Schenker et al., 2021) at weeks 2, 7 and 12 (Bjerring et al., 2020).

### 3.3 Risk of bias

**Fig. 2:**
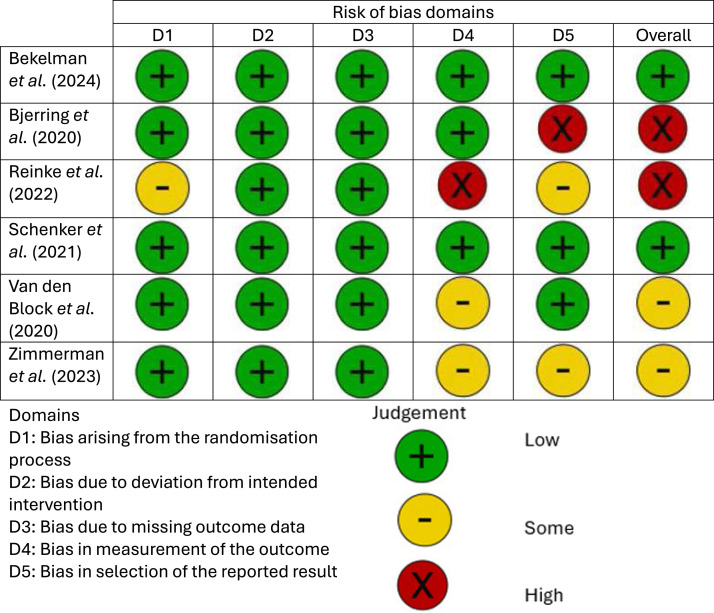
Risk of Bias Assessment (RoB 2).

Risk of bias scores for included studies are summarised in Fig. 2. The first three domains of the RoB 2 were rated as low for all studies, except one (Reinke et al., 2022). Assessment of the fourth and fifth domains revealed a mixture of 'low concerns' to ‘high concerns’ across all studies. Two studies had a ‘low’ risk of bias (Bekelman et al., 2024; Schenker et al., 2021), two had 'some concerns' (Van den Block et al., 2020; Zimmermann et al., 2023), and two had 'high concerns' (Bjerring et al., 2020; Reinke et al., 2022).

### 3.4 Interventions

The interventions varied in delivery frequency and duration. Three interventions were delivered monthly for 3 months (Schenker et al., 2021), monthly for 6 months (Zimmermann et al., 2023), or monthly for 12 months (Van den Block et al., 2020). Two studies reported delivering the intervention over 3 months (Bekelman et al., 2024; Reinke et al., 2022). One reported delivering the intervention at weeks 1, 6, and 11 (Bjerring et al., 2020). Of the six studies, three reported on intervention fidelity, which was good in one study (Bekelman et al., 2024), assessed but did not present fidelity findings (Reinke et al., 2022), or compromised by low adherence to the full intervention dose (Schenker et al., 2021).

All six studies referenced physical symptom assessment and management, although this was proxy measured by health professionals in one study (Van den Block et al., 2020). Other components of the interventions included a focus on symptom education (Reinke et al., 2022), person-centred care planning (Reinke et al., 2022), psychosocial (Bekelman et al., 2024), or social support needs (Bjerring et al., 2020; Zimmermann et al., 2023), and psychological (Zimmermann et al., 2023) or emotional support needs (Schenker et al., 2021). Nutrition needs were a component mentioned in the Bjerring et al. (2020) study.

### 3.5 Patient quality of life

Patient quality of life was examined in four studies, using four different measures (Bekelman et al., 2024; Bjerring et al., 2020; Schenker et al., 2021; Zimmermann et al., 2023). In one, an adequately-powered, multi-site, randomised control trial that consisted of a phone intervention with a nurse addressing symptoms and a social worker providing structured counselling, researchers demonstrated a statistically-significant improvement in quality of life at 6 months in the intervention group compared to usual care (Bekelman et al., 2024). One of the four studies was an adequately-powered multi-site randomised control trial that found no difference in quality of life scores (Schenker et al., 2021), and researchers in two single-site randomised control trials reported statistically non-significant improvements in quality of life (Bjerring et al., 2020; Zimmermann et al., 2023). Schenker et al*.* (2021) conducted a multi-site, randomised control trial and noted non-significant poorer outcomes in the intervention group at 6 months. The two single-site, randomised control trials that reported statistically non-significant improvements in quality of life were underpowered (Bjerring et al., 2020; Zimmermann et al., 2023), with one halted due to the COVID-19 pandemic (Zimmermann et al., 2023). Bjerring et al*.* (2020) reported a positive improvement trend towards global health-related quality of life scores in the intervention group that did not reach statistical significance. Similarly, Zimmerman et al. (2023) found that there was less deterioration in quality of life at 6 months in the intervention group, compared to the usual care group, but the difference was not statistically significant.

### 3.6 Patient symptom burden

In five studies, researchers reported the effects of nurse-led palliative care needs assessments on patient symptom burden compared to usual care. These assessments were reported by patients in four studies (Bekelman et al., 2024; Bjerring et al., 2020; Schenker et al., 2021; Zimmermann et al., 2023) and by health professional proxy measures in one study (Van den Block et al., 2020). One multi-site randomised control trial utilising a nurse-led and social worker-led intervention demonstrated a statistically-significant reduction in depression and anxiety in the intervention group compared to usual care (Bekelman et al., 2024). Two of the four studies found no difference in symptom burden scores (Bjerring et al., 2020; Zimmermann et al., 2023), and two studies reported statistically non-significant improvements in symptoms (Schenker et al., 2021; Van den Block et al., 2020). The two studies (Schenker et al., 2021; Van den Block et al., 2020) that found no significant difference in symptom burden scores were adequately-powered, multi-site, randomised control trials. Van den Block et al*.* (2020) reported no difference in the total symptom burden score compared to the control group, and Schenker et al. (2021) also reported no difference in symptom burden in individuals receiving the intervention. The underpowered, single-site randomised control trials reported statistically non-significant improvements in symptoms (Bjerring et al., 2020; Zimmermann et al., 2023). Zimmerman et al. (2023) noted a positive improvement trend at 6 months, and Bjerring et al*.* (2020) reported that the symptom scales generally displayed lower scores in individuals belonging to the intervention group at 12 weeks (no *p*-value stated).

### 3.7 Unplanned readmissions and length of stay

Bekelman et al. (2024) reported the unplanned hospitalisation rate, and no studies reported length of stay data. Bekelman et al*.* (2024) found there was no difference between the intervention group and the usual care groups amongst participants who had been hospitalised at 6 months.

## 4 Discussion

Despite one non-oncological randomised control trial demonstrating statistically-significant improvements in quality of life, depression, and anxiety (Bekelman et al., 2024), most of the findings were in oncology patients and revealed either no difference or statistically non-significant improvements in quality of life and symptom burden in this population. Non-statistically significant improvements may be due to studies being underpowered (Bjerring et al., 2020; Zimmermann et al., 2023), while the absence of differences in symptom burden and quality of life may be attributed to the intervention not being intensive enough to be effective or of low fidelity (Schenker et al., 2021), testing it in a group with little need (Schenker et al., 2021), or the intervention not being tailored to the context (Van den Block et al., 2020). Furthermore, the study designs were generally of poor quality, given that only two studies’ risk of bias was graded as low (Bekelman et al., 2024; Schenker et al., 2021), and one of these being in non-oncological patients (Bekelman et al., 2024). Limitations of the eligible studies included designs that precluded blinding of the participants, as the interventions were distinctive and required participants to be aware of their allocation (Schenker et al., 2021; Van den Block et al., 2020), and questionable sample size calculations for detecting differences in the quality of life endpoint (Bjerring et al., 2020). When we took these findings together, we found that there was a paucity of high-quality evidence on the effectiveness of nurse-led palliative care needs assessments on patient quality of life and symptom burden.

Although our findings on oncological patients and deceased aged-care residents revealed either no difference or non-statistically significant improvements in patient outcomes, a previous systematic review illustrated the potential of nurse-led care in producing equal or better outcomes for patient health-related quality of life and symptom burden compared to physician-led care for managing chronic conditions (Chan et al., 2018). However, the review conducted by Chan et al. (2018) did not focus on nurse-led care with the intention to identify patients in need of palliative care or evaluate needs for triage and tailored planning. Further, a systematic review of controlled trials (Carey et al., 2012) reported that most oncological intervention trials addressing unmet needs either reported no effect or limited effect and may have resulted from problems with intervention intensity, low statistical power, or sample selection, similar to the studies in the current review. Oncological studies that have tested targeted and tailored interventions for a group identified as having unmet needs or reduced quality of life have also demonstrated disappointing findings (Catherine et al., 2020; Girgis et al., 2009).

The researchers who deployed a nurse and social worker-led team found they made statistically-significant improvements in quality of life, depression, and anxiety (Bekelman et al., 2024). This differs from other non-oncological palliative care studies in addressing quality of life and depression (Chyr et al., 2022). However, the improvements may have been attributed to the use of collaborative care, a nurse as well as a social worker who had a key role in structured counselling and integrating information into ongoing outpatient care (Bekelman et al., 2024).

The remaining studies were focused on oncological patients, highlighting a gap in evidence regarding the impact of nurse-led palliative care needs assessment on patient outcomes in non-oncological illnesses. This gap is concerning, given the rise in patients living longer with chronic non-oncological illnesses, who experience higher hospital mortality and have a prevalence of palliative care needs similar to patients with cancer (Bandeali et al., 2020). Referral to specialist palliative care for individuals with non-oncological illnesses, such as heart failure, lags behind cancer, with referrals primarily based on disease-specific criteria rather than needs-based criteria (Chang et al., 2022). Consequently, referrals occur later in the illness trajectory, leading to missed opportunities to address palliative care concerns based on individual needs (Ye et al., 2024).

There are key considerations arising from this review for future research. Firstly, future research, involving interventions that are co-designed with key stakeholders and targets individuals with unmet needs, requires careful evaluation. A key consideration to furthering research in this area and informing intervention design is to examine what level of needs predicts poor quality of life and to differentiate between which needs can be met by the health system and which needs cannot, such as information needs that may, in fact, reflect a desire for certainty, rather than for information (Carey et al., 2012). Secondly, given the prevalence of palliative care needs in patients with non-oncological illnesses, there is a clear need for future researchers to explore how these needs can be screened for effectively and addressed in individuals with variable illness trajectories, prognoses, and treatment options (Bandeali et al., 2020). Interventions should be tailored and flexible to the local context, with a focus on a single, targeted component (Van den Block et al., 2020). A greater understanding of the appropriate intervention dose is needed (Schenker et al., 2021), and the outcome measures should be closely aligned to the intervention, as well as clinically-relevant (Bjerring et al., 2020; Van den Block et al., 2020). The improvements in patient outcomes demonstrated in the Bekelman et al. (2024) study warrant future research to further evaluate the implementation of the nurse-led and social worker-led collaborative care model. Researchers who address these gaps have the potential to enable more accessible healthcare for individuals with life-limiting oncological and non-oncological illnesses, with important implications for health policy and planning.

### 4.1 Strengths and limitations

The strengths of this review include the prospective protocol registration with PROSPERO and the inclusion of randomised control trials reporting the findings of the primary analysis exclusively. Covidence® was used for screening and data extraction, which enabled independent blinded review with reduced bias. A limitation of this review is the low quality of the included studies, given that only two studies’ risk of bias was graded as low, and so findings have to be interpreted with caution. There was a restriction to studies published in English, potentially overlooking global nuances.

## 5 Conclusion

Despite evidence from another systematic review that nurse-led services demonstrated equivalent or better outcomes for patient health-related quality of life and symptom burden (Chan et al., 2018), we demonstrated conflicting evidence on the effectiveness of nurse-led palliative care needs assessments. We found heterogeneity in needs assessment tools and outcome measures. Future research is crucial to determine what level of needs predicts poor quality of life and which needs can be met by the healthcare system. This will inform interventions for patients with life-limiting oncological and non-oncological illnesses that is tailored to their local context and evaluated using outcome measures that are clinically-relevant.

## Funding

This research did not receive any specific grant from funding agencies in the public, commercial, or not-for-profit sectors. However, a research assistant salary was partially funded through an internal University of the Sunshine Coast Early Career Research SPARK Grant.

## CRediT authorship contribution statement

**Gursharan K. Singh:** Writing – review & editing, Writing – original draft, Formal analysis, Data curation, Conceptualization. **Claudia Virdun:** Writing – review & editing, Formal analysis, Data curation, Conceptualization. **Megan Rattray:** Writing – review & editing, Formal analysis, Data curation. **Roslyn Prichard:** Writing – review & editing, Funding acquisition, Formal analysis. **Rochelle Wynne:** Writing – review & editing, Data curation, Conceptualization.

## Declaration of competing interest

The authors declare that they have no known competing financial interests or personal relationships that could have appeared to influence the work reported in this paper.

## Appendix Supplementary materials

Image, application 1
