# Supplementary material for: The effectiveness of nurse-led palliative care needs assessment on patients’ quality of life and symptom burden: a systematic review
Source: Int J Nurs Stud Adv. 2025 May 1;8:100343. doi: 10.1016/j.ijnsa.2025.100343 (PMC12135387; doi:10.1016/j.ijnsa.2025.100343)
Supplement: Supplementary file 1 [file mmc1.docx]

**Appendix A: Search Strategy**

- Medline

| S1 | (MH "Bereavement+") | 15,106 |
| --- | --- | --- |
| S2 | (MH "Hospices") OR (MH "Hospice Care") OR (MH "Hospice and Palliative Care Nursing") | 14,703 |
| S3 | (MH "Terminal Care+") | 56,750 |
| S4 | "end-of-life" | 43,966 |
| S5 | (MH "Terminally Ill") | 6,802 |
| S6 | (MH "Palliative Medicine") OR (MH "Palliative Care") | 62,863 |
| S7 | "palliative therapy" | 37,191 |
| S8 | "need*" | 2,426,074 |
| S9 | (MH "Nurses+") | 97,908 |
| S10 | (MH "Practice Patterns, Nurses") OR "nurse led" | 7,500 |
| S11 | "nurse led or nurse-led or nurse managed or nurse delivered or nurse based or nurse" | 48,374 |
| S12 | S1 OR S2 OR S3 OR S4 OR S5 OR S6 OR S7 | 147,195 |
| S13 | S9 OR S10 OR S11 | 104,604 |
| S14 | S8 AND S12 AND S13 | 758 |

- CINAHL

| S1 | (MH "Terminal Care+") | 72,085 |
| --- | --- | --- |
| S2 | MH "Bereavement+") | 15,921 |
| S3 | (MH "Hospice Patients") OR (MH "Hospice and Palliative Care Nursing") OR (MH "Hospice Care") OR (MH "Hospices") | 12,463 |
| S4 | "end-of-life" | 33,667 |
| S5 | (MH "Terminally Ill Patients+") | 12,644 |
| S6 | (MH "Palliative Medicine") OR (MH "Palliative Care") | 41,488 |
| S7 | "palliative therapy" | 29,141 |
| S8 | Need* | 827,476 |
| S9 | (MH "Nurses+") | 224,220 |
| S10 | "nurse led" | 5,575 |
| S11 | "nurse led or nurse-led or nurse managed or nurse delivered or nurse based or nurse" OR (MH "Nurse-Managed Centers") | 2,464 |
| S12 | S1 OR S2 OR S3 OR S4 OR S5 OR S6 OR S7 | 103,752, |
| S13 | S9 OR S10 OR S11 | 230,053 |
| S14 | S8 AND S12 AND S13 | 1,204 |

- PubMed

| 1 | "Terminal Care"[Mesh] OR "Hospices"[Mesh] OR "Palliative Care"[Mesh] OR "Palliative Medicine"[Mesh] | 109,845 |
| --- | --- | --- |
| 2 | end of life | 1,126,529 |
| 3 | bereave* | 12,033 |
| 4 | terminally ill | 11,276 |
| 5 | palliative* | 125,068 |
| 6 | ("Terminal Care"[Mesh] OR "Hospices"[Mesh] OR "Palliative Care"[Mesh] OR "Palliative Medicine"[Mesh]) OR "end of life" OR bereave* OR "terminally ill" OR palliative*) | 1,248,320 |
| 7 | needs assessment | 90,517 |
| 8 | unmet* | 43,605 |
| 9 | need* | 2,390,921 |
| 10 | "needs assessment" OR unmet* OR need* | 2,400,670 |
| 11 | "Nurses"[Mesh] | 97,770 |
| 12 | nurse-led | 4,972 |
| 13 | ("Nurses"[Mesh]) OR nurse-led) | 101,995 |
| 14 | ("Terminal Care"[Mesh] OR "Hospices"[Mesh] OR "Palliative Care"[Mesh] OR "Palliative Medicine"[Mesh]) OR "end of life" OR bereave* OR "terminally ill" OR palliative*)AND ("needs assessment" OR unmet* OR need*) AND ("Nurses"[Mesh]) OR (nurse-led)) | 1,177 |

- Embase

| 1 | 'palliative therapy'/exp OR 'palliative therapy' | 140,448 |
| --- | --- | --- |
| 2 | 'terminal care'/exp OR 'terminal care' | 85,035 |
| 3 | bereave* | 16,702 |
| 4 | hospice* | 71,167 |
| 5 | 'end of life'/exp OR 'end of life' | 47,667 |
| 6 | 'terminally ill patient'/exp | 9,515 |
| 7 | palliative* | 226,167 |
| 8 | #1 OR #2 OR #3 OR #4 OR #5 OR #6 OR #7 | 332,907 |
| 9 | 'nurse led' | 8,303 |
| 10 | 'nurse delivered' | 549 |
| 11 | 'nurse'/exp | 215,673 |
| 12 | #9 OR #10 OR #11 | 219,282 |
| 13 | 'needs assessment'/exp | 26,986 |
| 14 | 'need' | 1,576,856 |
| 15 | #13 OR #14 | 1,595,848 |
| 16 | #8 AND #12 AND #15 | 2,860 |

MH: Mesh Heading
